# Nanoscale clustering of mycobacterial ligands and DC-SIGN host receptors are key determinants for pathogen recognition

**DOI:** 10.1126/sciadv.adf9498

**Published:** 2023-05-19

**Authors:** Albertus Viljoen, Alain Vercellone, Myriam Chimen, Gérald Gaibelet, Serge Mazères, Jérôme Nigou, Yves F. Dufrêne

**Affiliations:** ^1^ Louvain Institute of Biomolecular Science and Technology, UCLouvain, Croix du Sud, 4-5, bte L7.07.07., B-1348 Louvain-la-Neuve, Belgium.; ^2^ Institut de Pharmacologie et de Biologie Structurale (IPBS), Université de Toulouse, CNRS, Université Toulouse III - Paul Sabatier (UPS), Toulouse, France.

## Abstract

The bacterial pathogen *Mycobacterium tuberculosis* binds to the C-type lectin DC-SIGN (dendritic cell–specific intercellular adhesion molecule 3-grabbing nonintegrin) on dendritic cells to evade the immune system. While DC-SIGN glycoconjugate ligands are ubiquitous among mycobacterial species, the receptor selectively binds pathogenic species from the *M. tuberculosis* complex (*MTBC*). Here, we unravel the molecular mechanism behind this intriguing selective recognition by means of a multidisciplinary approach combining single-molecule atomic force microscopy with Förster resonance energy transfer and bioassays. Molecular recognition imaging of mycobacteria demonstrates that the distribution of DC-SIGN ligands markedly differs between *Mycobacterium bovis* Bacille Calmette-Guérin (BCG) (model *MTBC* species) and *Mycobacterium smegmatis* (non-*MTBC* species), the ligands being concentrated into dense nanodomains on *M. bovis* BCG. Upon bacteria-host cell adhesion, ligand nanodomains induce the recruitment and clustering of DC-SIGN. Our study highlights the key role of clustering of both ligands on *MTBC* species and DC-SIGN host receptors in pathogen recognition, a mechanism that might be widespread in host-pathogen interactions.

## Teaser

Glycoconjugate ligand clusters on a bacterial pathogen drives selective recognition by host pathogen recognition receptor.

## INTRODUCTION

A number of the most important pathogens, including the enveloped viruses HIV (*1*–*3*), Ebola (*4*), dengue (*5*), and severe acute respiratory syndrome coronavirus 2 (*6*, *7*), and the bacteria *Helicobacter pylori* (*8*), *Klebsiella pneumoniae* (*9*), and *Mycobacterium tuberculosis* (*Mtb*) (*10*, *11*) are recognized by the C-type lectin pattern recognition receptor (PRR) DC-SIGN (dendritic cell–specific intercellular adhesion molecule 3-grabbing nonintegrin), in a key step leading to their internalization by antigen-presenting dendritic cells (DCs). Some of these microbes exploit DC-SIGN to induce an anti-inflammatory response to evade the immune response (*10*, *12*–*14*). A notable example is *Mtb* (*10*, *15*–*17*), the causative agent of human tuberculosis, which, until the coronavirus disease 2019 pandemic, was the global leading cause of death from a single infectious agent, ranking above HIV/AIDS (*18*).

DC-SIGN binds in a Ca^2+^-dependent manner to diverse l-fucosylated glycans, N-linked high d-mannose oligosaccharides (*19*, *20*), and, in the case of mycobacteria, α-(1 → 2)-oligomannosides and α-glucans (*10*, *11*, *21*–*23*). Because of the tetrameric state of the receptor, the C-terminal carbohydrate recognition domains can engage in multivalent interactions, thereby functionally amplifying the receptor avidity for its ligands (*24*, *25*). Moreover, DC-SIGN has been observed in multimolecular clusters on immature DCs ranging in size from ~200 nm to ~1 μm in diameter (*26*–*29*). In addition, intrinsic flexibility within the DC-SIGN tetramer may permit individual lectin domains to bind sparsely spaced ligands (*30*).

While nonopsonized *Mtb* enters macrophages through binding to the complement or mannose receptors, DCs mainly recognize the pathogen through DC-SIGN (*11*). Moreover, among mycobacteria, DC-SIGN binds selectivity to *Mtb* complex (*MTBC*) species, the closest relatives of *Mtb* (*21*). Yet, the envelopes of mycobacteria within and outside of the *MTBC* are rich in mannose- or glucose-based glycans or glycoconjugates (*31*–*34*) that are likely to be DC-SIGN ligands. Of these, purified mannose-capped lipoarabinomannan (ManLAM) (*11*, *22*), lipomannan (LM) (*21*), phosphatidylinositol hexamannosides (PIM_6_) (*21*, *35*), mannoproteins (*21*), and α-glucan (*23*) were shown to bind DC-SIGN, but none of these are specifically produced by *MTBC* species. *Mtb* deletion mutants that do not produce ManLAM (*36*), PIM_6_ (*37*), both molecules (*37*), or mannoproteins (*21*, *34*) retained their ability to bind DC-SIGN at wild-type levels.

Consequently, a critical, yet currently unsolved, issue is that while DC-SIGN ligands are redundant on mycobacterial cell surfaces, irrespective of pathogenic and nonpathogenic species, the receptor selectively binds pathogenic *MTBC* species. As the difference in mycobacterial species recognition by DC-SIGN does not primarily rely on the type of glycoconjugates they produce, other unknown mechanisms must be at play. Here, we address this using single-molecule atomic force microscopy (AFM) (*38*), combined with Förster resonance energy transfer (FRET) and bioassays. We find that DC-SIGN ligands are concentrated into densely arranged nanodomains on the surface of *Mycobacterium bovis* Bacille Calmette-Guérin (BCG) (used as a model *MTBC* species), while they are essentially randomly distributed on *Mycobacterium smegmatis* (a non-*MTBC* species). This is accompanied by the presence of large membrane-expressed DC-SIGN clusters upon adhesion of bacteria to host cells and by adhesion-induced recruitment of DC-SIGN. Our findings demonstrate that the clustering of mycobacterial ligands and the clustering of host DC-SIGN are key determinants for pathogen recognition, therefore rationalizing, at the molecular level, the highly selective recognition of *MTBC* by DC-SIGN. This mechanism might be widespread among pathogen-immune cell interactions involving DC-SIGN but possibly also other PRRs, with consequences for the modulation of the immune response during infection.

## RESULTS

### DC-SIGN ligands are surface-localized and available for binding on *MTBC* and non-*MTBC* members

**Fig. 1.**
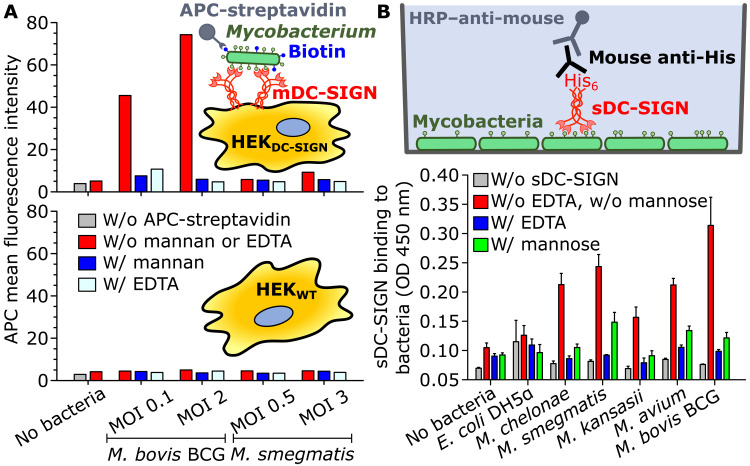
Cell membrane DC-SIGN discriminates between *MTBC* and non-*MTBC* members, although they nondifferentially express surface ligands available for binding interactions. (**A**) HEK 293 cells heterologously expressing mDC-SIGN binds *M. bovis* BCG (model *MTBC* species) but not *M. smegmatis* (model non-*MTBC* species). Biotinylated mycobacteria were incubated with HEK cells that do not (HEK_WT_) or that do express DC-SIGN (HEK_DC-SIGN_). Bound bacteria were labeled with allophycocyanin (APC)–conjugated streptavidin and detected by flow cytometry. The data shown are representative of two independent experiments. The bars show the mean fluorescence intensity. Specificity of binding was confirmed through blocking with mannan (3 mg ml^−1^) or EDTA (2 mM). (**B**) Microtiter plate-bound assay to test the binding of a recombinant soluble form of the extracellular domain of DC-SIGN (sDC-SIGN) to diverse mycobacterial species. Bars indicate means, and error bars show the SE. The data shown are representative of four independent experiments. Specificity of binding was tested by adding 100 mM mannose or 5 mM EDTA. *E. coli* DH5α produces a lipopolysaccharide unlikely to bind DC-SIGN (*41*, *42*). MOI, multiplicity of infection; OD, optical density; HRP, horseradish peroxidase.

We first tested the extent to which the *MTBC* species model *M. bovis* BCG and the non-*MTBC* model nonpathogenic species *M. smegmatis* are recognized by DC-SIGN expressed at the membrane (mDC-SIGN) of a human embryonic kidney cell line (HEK_DC-SIGN_). Using flow cytometry analysis, we found that *M. bovis* BCG bound to HEK_DC-SIGN_ cells in a multiplicity-of-infection (MOI)–dependent fashion, while it did not bind to wild-type HEK cells (HEK_WT_) (Fig. 1A and fig. S1). As expected, *M. bovis* BCG binding to HEK_DC-SIGN_ cells was inhibited by mannan [a high-affinity DC-SIGN ligand (*24*, *39*)] and by the divalent cation chelator EDTA. In contrast, *M. smegmatis* did not bind the HEK_DC-SIGN_ cells appreciably beyond background control levels (Fig. 1A). These results are in line with previous work in which an mDC-SIGN–expressing HELA cell line bound *MTBC* species selectively among phylogenetically diverse mycobacteria (*21*).

Intriguingly, mDC-SIGN binds purified forms of LM, PIM_6_, mannoproteins, and α-glucan (*21*, *23*, *35*, *37*), all present in *M. smegmatis*, while it does not recognize *M. smegmatis*. A possible explanation could be that these potential ligands are located within deeper layers of the envelope and masked from interactions with DC-SIGN. To test this hypothesis, we first produced a recombinant soluble form of the extracellular domain of DC-SIGN [sDC-SIGN; (*40*)], which presented as a tetramer and was functional in binding known ligands, including ManLAM, LM, PIM_2_, and PIM_6_ (fig. S2). Next, we developed a binding assay using sDC-SIGN and microtitre plates coated with various bacterial species, including non-*MTBC* species (*Mycobacterium chelonae*, *Mycobacterium kansasii*, *Mycobacterium avium*, and *M. smegmatis*), the *MTBC* model *M. bovis* BCG, and *Escherichia coli* DH5α as a control bacterium (Fig. 1B) (*41*, *42*). Although the highest levels of sDC-SIGN binding occurred for *M. bovis* BCG, all mycobacterial species substantially bound the extracellular domain of the receptor, strongly supporting the notion that DC-SIGN ligands are surface-exposed on mycobacterial species both within and outside of the *MTBC*. Therefore, there must be a currently unknown mechanism explaining why mDC-SIGN does not bind non-*MTBC* species despite the presence of ligands on their surface.

### Mechanical strength of single sDC-SIGN–ligand complexes on living mycobacteria

**Fig. 2.**
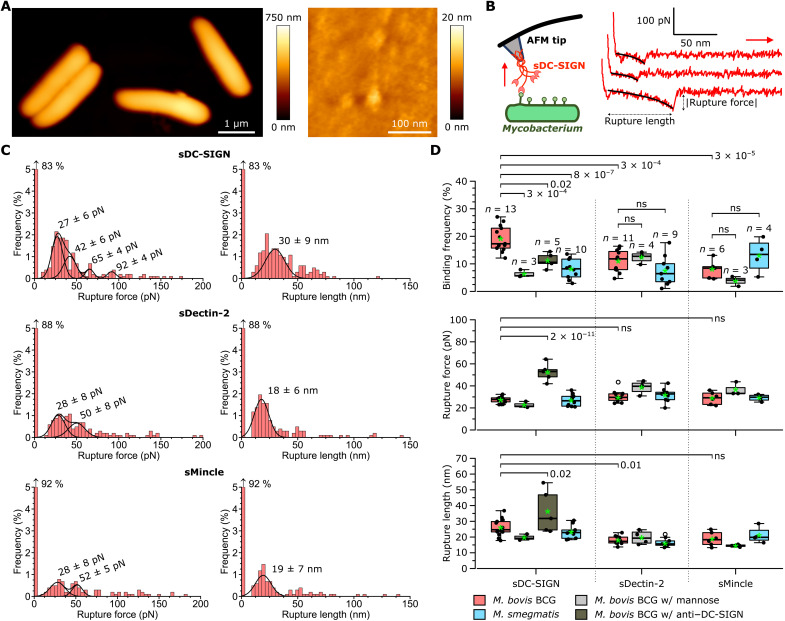
Binding probability but not binding strength of single sDC-SIGN–ligand complexes is greater for *M. bovis* BCG than for *M. smegmatis*. (**A**) AFM height images of single *M. bovis* BCG cells (right, high-resolution image recorded on top of a single bacterium). (**B**) In single-molecule force spectroscopy, an AFM tip bearing a C-type lectin is lowered onto a living mycobacterial cell. Retracting the tip from the cell surface leads to the rupture of single receptor-ligand complexes (*38*). Right: Representative retraction force profiles (red) obtained between sDC-SIGN–modified tips and *M. bovis* BCG are shown. The black line shows a worm-like chain model fit of the data. (**C**) Representative rupture force (left) and length (right) histograms generated from 1024 force curves recorded on a single *M. bovis* BCG cell using tips functionalized with either sDC-SIGN, (top), sDectin-2 (middle), or sMincle (bottom). The leftmost bar in each histogram indicates the percentage of curves showing no binding. Means ± SD values are indicated above each gaussian peak. (**D**) Boxplots of the mean binding frequencies, rupture forces, and rupture lengths measured for multiple cells (*n* values in the top panel). Specific binding was inhibited by 100 mM mannose or anti–DC-SIGN (10 μg ml^−1^). In boxplots, thick bars represent the medians; green asterisks represent the means; bottoms and tops of boxes represent the first and third quartiles, respectively; and whiskers represent the range. Differences in sample distributions were evaluated using Tukey’s post hoc multiple comparisons test with α = 0.05. *P* values are indicated to the right of comparison braces. ns, nonsignificant.

We wondered whether the binding strength between DC-SIGN receptors and their glycoconjugate ligands might differ between *MTBC* and non-*MTBC* species. To test this hypothesis, we used AFM, a multifunctional nanotechnique allowing the probing of the surface ultrastructure and molecular interactions of living bacterial cells, in a way that is inaccessible to conventional microscopy and biochemical assays (*43*–*45*). Figure 2A shows topographic images of whole *M. bovis* BCG cells (left) and the high-resolution surface of one such bacterium (right). It evidences a cell surface roughness of 0.6 ± 0.1 nm (means ± SD from seven different cells), indicating that the ultrastructure on the bacterial surface is very flat, as typically observed for mycobacteria (*46*–*49*), a factor that enhances the area available for adhesion (*50*).

To quantify the strength of single DC-SIGN–ligand complexes, we used single-molecule force spectroscopy (SMFS), in which AFM tips functionalized with sDC-SIGN, carrying an N-terminal immunoglobulin G (IgG)–Fc fusion for optimal orientation, were used to generate multiple force-distance (*FD*) curves across living mycobacteria. Analyzing *FD* curves (Fig. 2B, right) yielded binding frequencies (i.e., the percentage of curves with binding events among the total number of curves recorded per cell), rupture forces (bond strengths), and rupture lengths (extension at which the complex ruptures) (Fig. 2, C and D). sDC-SIGN binds ligands on *M. bovis* BCG with a frequency of 19 ± 5% (means ± SD, from a total of 11,776 curves from *n* = 13 cells). The rupture forces adopted a multimodal distribution with a first and dominant peak representing the rupture of single bonds, followed by minor peaks centered at multiples of the first peak (Fig. 2C and fig. S3). From a total of 13 different cells, we obtained an average rupture force for single complexes of 27 ± 3 pN (Fig. 2D). This rupture force is in the range of those reported for other lectin-carbohydrate interactions (*51*–*55*). The force profiles were well fitted by the worm-like chain model of biopolymer extension (Fig. 2B), in agreement with the stretching of receptor-ligand complexes. Our tip functionalization protocol ensures a minimal number of PRRs at the tip apex (*56*, *57*), which favors formation of single molecular complexes. Yet, parallel bond formation was observed with larger rupture forces (fig. S3), most likely arising from the tetrameric state (fig. S4) of the extracellular domain of DC-SIGN (*25*, *58*, *59*) that allows multivalent interactions with up to four carbohydrate moieties. Single complexes extended over an average length of 26 ± 5 nm (from a total of 11,776 curves from *n* = 13 cells; Fig. 2, C and D).

To prove the specificity of the interactions, we first used bare silicon nitride tips. These bound with a negligible frequency (~3%) to the mycobacterial surfaces (fig. S5). In addition, mannose-blocking reduced binding threefold (6 ± 1%, from a total of 3072 curves from three cells) but had virtually no effect on rupture forces and rupture lengths (Fig. 2D). Last, injecting a polyclonal antibody raised against a sequence within the C-terminal of DC-SIGN where its C-type lectin domain is located also caused a substantial decrease in binding frequency (11 ± 3%, from a total of 5120 curves from *n* = 5 cells).

We then asked whether *M. bovis* BCG ligands interact in a similar way with soluble forms of the extracellular domains of two related C-type lectin PRRs, Dectin-2 (DC-associated C-type lectin-2; sDectin-2) and Mincle (macrophage inducible Ca^2+^-dependent lectin; sMincle). The structures and mycobacterial-glycoconjugate binding specificities of these PRRs diverge from DC-SIGN; Mincle recognizes all mycobacterial species via a plethora of lipid ligands (*60*, *61*), whereas Dectin-2, having ManLAM as the sole ligand, recognizes slow-growing mycobacteria only (*62*, *63*). Single molecular complexes for both receptors ruptured under similar forces (31 ± 5 pN, *n* = 11,264 total curves from 11 cells for sDectin-2 and 29 ± 6 pN, *n* = 6,144 total curves from 6 cells for sMincle) to those formed by sDC-SIGN (Fig. 2, C (middle and bottom) and D). Bimodal distributions were observed for these receptors (Fig. 2C and fig. S6), which may be accounted for by their monomeric and dimeric states (*64*, *65*). Contrasting with sDC-SIGN, complexes with these PRRs ruptured at shorter extension lengths (18 ± 3 nm for sDectin-2 and 19 ± 4 nm for sMincle), in agreement with the smaller sizes of their extracellular domains (see Materials and Methods). Notably, binding frequencies for both receptors were significantly lower than for sDC-SIGN at 11 ± 4% for sDectin-2 and only 8 ± 3% for sMincle, implying that DC-SIGN is the C-type lectin PRR receptor that most readily interacts with pathogen-associated molecular patterns on the *M. bovis* BCG surface.

Notably, sDC-SIGN bindsligands on the *M. smegmatis* surface with a frequency of 8 ± 3%, which is about twofold lower than in *M. bovis* BCG (*n* = 10 cells; Fig. 2D), and there were no differences in rupture forces and lengths (Fig. 2D). In addition, as expected, Dectin-2 appeared to interact the weakest with the molecules exposed on the surface of *M. smegmatis*.

### sDC-SIGN binds *M. bovis* BCG and *M. smegmatis* ligands with similar kinetics

**Fig. 3.**
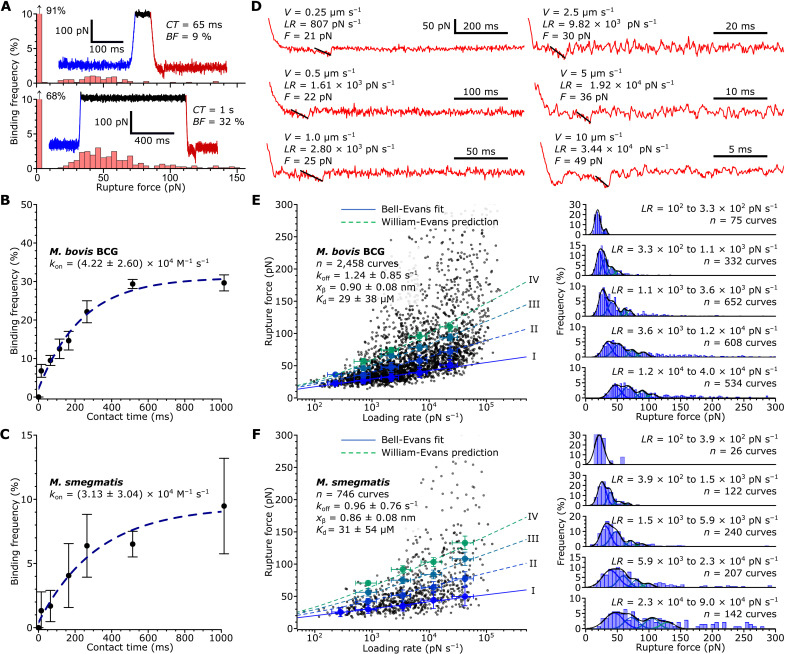
sDC-SIGN binds to ligands on *M. bovis* BCG and *M. smegmatis* with similar kinetics. (**A**) Histograms of data obtained by probing *M. bovis* BCG with an sDC-SIGN–modified tip using contact times (*CT*) of 65 ms (top) and 1 s (bottom). The force-time curves in the insets contain approach (blue), contact pause (black), and retraction (red) portions. (**B** and **C**) Binding frequency (*BF*) versus *CT* scatterplots obtained for *M. bovis* BCG [(B), *n* = 3 cells] and *M. smegmatis* [(C), *n* = 3 cells] and associated pseudo–first-order kinetics fits. Means and SD are indicated by solid circles and error bars, respectively. (**D**) Six representative force-time retraction curves acquired at different tip retraction velocities (*V*). *LR*, force loading rate; *F*, rupture force. The slope (black line) at maximum molecular extension before rupture gives *LR*. (**E** and **F**) Dynamic force spectroscopy plots (left) and associated histogram plots (right) for *M. bovis* BCG [(E), *n* = 3 cells] and *M. smegmatis* [(F), *n* = 3 cells]. The solid blue line in the plots show the Bell-Evans fit of single-bond (I) data (*70*). The broken lines show predictions for uncooperative double (II), triple (III), and quadruple bonds (IV) made from the Bell-Evans fit parameters (*112*, *113*). *x*_β_ indicates the distance along the reaction coordinate to the transition between bound and unbound states. Means and SD are indicated by the large solid circles and error bars, respectively.

Next, we aimed to understand why *M. smegmatis* ligands are bound with a lower frequency by sDC-SIGN compared to *M. bovis* BCG. One explanation may be that sDC-SIGN binds ligands on *M. smegmatis* with a lower overall affinity. DC-SIGN affinity might differ for the different individual ligands, and their relative abundances are not clearly defined between *M. bovis* BCG and *M. smegmatis*, which, in addition, does not produce ManLAM (*66*). Using SMFS, binding kinetics parameters can be estimated (*67*, *68*) for ligands exposed in their native form on living bacteria. A pseudo–first-order kinetics analysis of the relationship between binding frequency and probe-bacterial surface contact time (*67*) allows estimating the kinetic on rate constant of a molecular interaction (*k*_on_; Fig. 3A). This yielded similar *k*_on_ values for DC-SIGN ligands on *M. bovis* BCG and *M. smegmatis* (4.2 ± 2.6) × 10^4^ M^−1^ s^−1^ (Fig. 3B) and (3.1 ± 3.0) × 10^4^ M^−1^ s^−1^ (Fig. 3C), respectively. The off-rate constant (*k*_off_) can be assessed (*69*, *70*) from dynamic force spectroscopy experiments, wherein the tip retraction speed is varied over a wide range of constant velocities to get force (*F*) versus loading rate (*LR*) plots (Fig. 3D). Analyses of *F* versus *LR* data using the Bell-Evans (*70*) model yielded *k*_off_ values of 1.2 ± 0.9 s^−1^ and 1.0 ± 0.8 s^−1^ for *M. bovis* BCG (Fig. 3E) and *M. smegmatis*, respectively (Fig. 3F), thus in a similar range. Derived *K*_d_ (dissociation constant) (= *k*_off_/*k*_on_) values for both species were ~30 μM, which agrees well with affinity values obtained by ensemble measurements for DC-SIGN (soluble recombinant extracellular fragment) interacting with synthetic high-mannose oligosaccharide ligands (*25*). These results show that *M. bovis* BCG and *M. smegmatis* exhibit similar DC-SIGN–ligand binding kinetics. This led us to hypothesize that the main factor defining differences in the overall mDC-SIGN binding properties is the spatial distribution of ligands across the bacterial surface, rather than the binding strength or kinetics.

### Ligand clusters cover most of the *M. bovis* BCG surface, while ligands are sparsely distributed on *M. smegmatis*

**Fig. 4.**
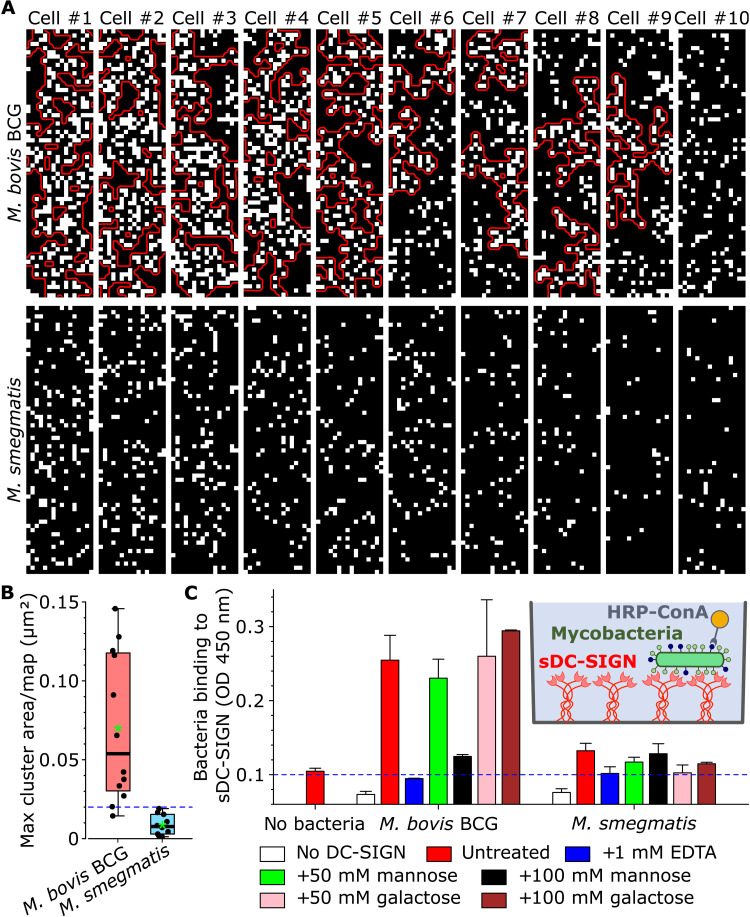
*M. bovis* BCG but not *M. smegmatis* exhibits large ligand clusters, which correlates with selective binding of *M. bovis* BCG by high-density immobilized sDC-SIGN. (**A**) Molecular recognition maps (0.25 μm by 1.0 μm) recorded using DC-SIGN–modified AFM tips. White and black pixels indicate the presence and absence of ligands. Red lines demarcate clusters with area greater than 0.02 μm^2^. (**B**) Boxplot of maximal cluster area measured per map. The difference in sample distributions was tested for significance using a one-tailed Mann-Whitney *U* test with *P* < 0.001. The thick bars in boxplots represent the median; the green asterisks represent the means; the bottoms and tops of the boxes represent the first and third quartiles, respectively; and the whiskers represent the range. (**C**) Microtiter plate assay showing that a high-density sDC-SIGN surface binds *M. bovis* BCG selectively. The data shown are representative of four independent experiments. Man, mannose; Gal, l-galactose. Means and SD are indicated by bars and error bars SD, respectively. OD, optical density.

To assess ligand surface distribution, we generated molecular recognition maps with sDC-SIGN–modified tips, wherein white and black pixels (16 nm–by–16 nm in size) indicate the presence or absence of a ligand (Fig. 4A). A dense clustered distribution of ligands was observed on *M. bovis* BCG, while they were essentially scattered on *M. smegmatis* (Fig. 4A). For a quantitative analysis, we defined a ligand cluster as any contiguous area containing at least two white pixels that are not separated by more than one black pixel, implying a maximal distance between two ligands of ~45 nm, which is roughly equivalent to the upper range nearest neighbor spacing of DC-SIGN molecules in lipid rafts on immature DCs (*26*, *27*). While *M. smegmatis* showed ligand clusters always smaller than 0.02 μm^2^ (*n* = 10 cells), *M. bovis* BCG featured ligand clusters that were much larger, ranging from 0.03 to 0.12 μm^2^ (interquartile range, *n* = 12 cells; Fig. 4B). Because a cluster area of 0.02 μm^2^ contains at least 40 ligands (based on a cluster density of ~2000 ligands per μm^2^), our results suggest that there may be an optimal threshold for efficient binding to mycobacterial ligands by cell membrane DC-SIGN.

Knowing that high surface density of DC-SIGN is required for efficient binding of particles such as viruses, whereas it is not for efficient binding of soluble ligands (*26*, *71*), we wondered whether a high density of receptors could be important for selective recognition of *MTBC* species. To test this hypothesis, we developed a binding assay using His-tagged sDC-SIGN coated onto Ni-chelate microplates at saturated levels (fig. S7) and incubated with *M. bovis* BCG or *M. smegmatis*. After washing, *M. bovis* BCG cells remained attached to the sDC-SIGN surface, while *M. smegmatis* did not (Fig. 4C). *M. bovis* BCG binding was specific to DC-SIGN as it was inhibited by EDTA and mannose, but not by galactose. These results suggest that the DC-SIGN density plays a role in the selective binding of *MTBC* species. In addition, it is conceivable that the forces exerted on DC-SIGN–ligand complexes, where whole bacteria are in solution while DC-SIGN is immobilized (Fig. 4C), are much greater than when the bacteria are immobilized, and the receptor is in solution (Fig. 1B). It is possible that the greater shear forces at play in the former configuration (Fig. 4C), which likely approximates the in vivo context, also contribute to differential binding.

### *M. bovis* BCG adhesion to mDC-SIGN–expressing host cells is higher and involves receptor clustering

**Fig. 5.**
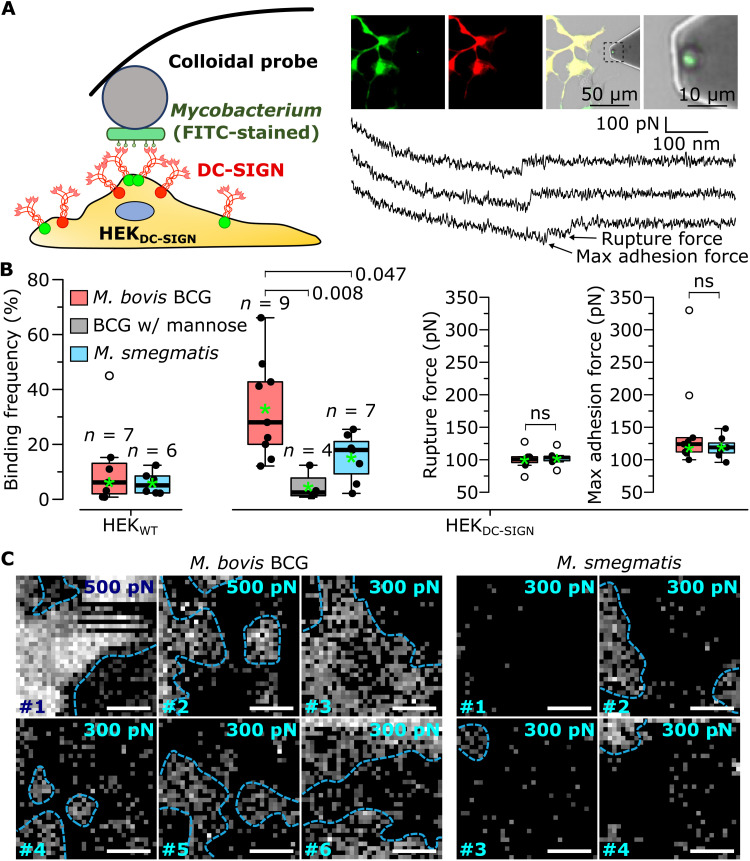
Single-cell force spectroscopy shows that adhesion to HEK cells expressing mDC-SIGN is enhanced in *M. bovis* BCG and involves mDC-SIGN clustering (**A**) Single-cell force spectroscopy measures the interaction forces between a mycobacterial probe and a HEK_DC-SIGN_ cell. Red and green circles represent enhanced green fluorescent protein (EGFP) and DsRed fused to the N termini of DC-SIGN. Top right: Epifluorescence and differential interference contrast images showing a single fluorescein isothiocyanate (FITC)–stained mycobacterium on a colloidal probe as well as HEK_WT_ (nonfluorescent) and HEK_DC-SIGN_ (green and red fluorescent) cells. Bottom right: Representative force curves obtained between a *M. bovis* BCG and a HEK_DC-SIGN_ cell. In curves showing serial rupture events (bottom), the maximum adhesion force sustained by parallel bonds is larger than the rupture force of a single molecular complex. (**B**) Boxplots of the mean binding frequencies, rupture forces, and rupture lengths measured for several bacterial–HEK cell pairs (*n* values indicated above the boxes). Blocking was done using 100 mM mannose. Thick bars represent the median; green asterisks represent the means; bottoms and tops of boxes represent the first and third quartiles, respectively; and whiskers represent the range. Differences in sample distributions were evaluated using either Tukey’s post hoc multiple comparisons test (binding frequencies) or one-tailed Mann-Whitney *U* tests with α = 0.05. *P* values are indicated to the right or on top of comparison braces. (**C**) Molecular recognition maps obtained with *M. bovis* BCG (left) or *M. smegmatis*–modified (right) probes and HEK_DC-SIGN_ cells. Each map represents an individual bacterium–HEK cell pair. White scale bars, 1 μm. The grayscale indicates the maximum adhesion force and ranges from 0 pN (black pixel) to the value indicated in the top right corner of each map.

Single-cell force spectroscopy (SCFS) (*38*), in which a single bacterial cell is bound to a colloidal AFM probe (Fig. 5A, left), allows the evaluation of force magnitudes and frequencies in bacterial-host cell adhesion. This method allowed us to measure the adhesion forces between single *M. bovis* BCG or *M. smegmatis* cells and HEK_DC-SIGN_ or HEK_WT_ (control) cells (Fig. 5A, top right). Like in SMFS, *FD* curves showed a nonlinear extension profile indicative of biomolecular extension (Fig. 5A, bottom right). Some curves exhibited serial unbinding events resulting from sequential unbinding of multiple bonds (*72*), with the largest peak being the maximum adhesion force, while the last peak yielded the rupture force of single molecular complexes (Fig. 5B, bottom right).

Average rupture forces of ~100 pN were measured for both species (Fig 5B), considerably greater than the SMFS values. This originates from the much higher *LR* used due to the considerably stiffer (*k* ≈ 0.1 N m^−1^) colloidal probe cantilevers and to the faster retraction speed (20 μm s^−1^) required to limit HEK cell membrane deformation. *M. bovis* BCG bound to the HEK_DC-SIGN_ cells with a frequency of 33 ± 18% (means ± SD, *n* = 9 bacterium-cell pairs), significantly greater than *M. smegmatis* (15 ± 9%, means ± SD, *n* = 7 bacterium-cell pairs; Fig. 5, B and C). In addition, ~25% of *M. bovis* BCG cells exhibited maximum adhesion forces of ~200 pN and greater, considerably above the population mean (117 ± 11 pN, *n* = 9 bacterium-cell pairs; Fig. 5B); this was not observed for *M. smegmatis.* Taking the adhesion force as a rough indicator of valency, on average, only one or two cellular receptors bound the *M. bovis* BCG probes. We assign this to the rapid probe velocity we used here, resulting in short contact times (~25 ms), which would limit the number of bonds that can form. In control experiments, we found that (i) injection of free mannose strongly reduced binding in HEK_DC-SIGN_ cells and (ii) HEK_WT_ cells poorly bound *M. bovis* BCG (Fig. 5B), indicating that mDC-SIGN expressed on HEK_DC-SIGN_ cells is surface exposed and fully functional and that it represents the main receptor for mycobacteria.

Notably, molecular recognition maps obtained with *M. bovis* BCG probes revealed stark segregation of DC-SIGN on the host cell surfaces, with clear DC-SIGN clusters surrounded by zones practically devoid of the receptor (Fig. 5C). This phenomenon was much less apparent with *M. smegmatis* probes. These results show that a clustered distribution of DC-SIGN must play a role in the selective recognition of *MTBC* species.

### Large ligand clusters on *M. bovis* BCG correlates with mDC-SIGN recruitment

**Fig. 6.**
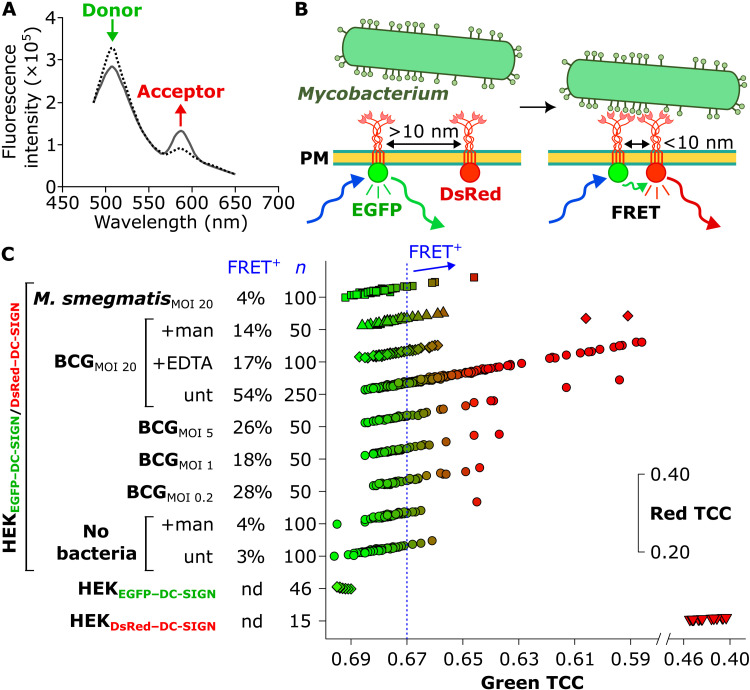
Förster resonance energy transfer reveals that *M. bovis* BCG adhesion to HEK_DC-SIGN_ induces DC-SIGN recruitment. (**A**) Fluorescence emission for EGFP (donor) and DsRed (acceptor) under excitation at 476 nm. Because of the FRET effect, peak fluorescence emission by GFP (at λ ≈ 500 nm) is reduced, while the fluorescence of DsRed is induced (peak emission at λ ≈ 600 nm). (**B**) Proposed mechanism of FRET between EGFP–DC-SIGN and DsRed–DC-SIGN induced by binding of mycobacteria exhibiting dense ligand clusters. For simplicity, only one EGFP or DsRed is depicted per DC-SIGN tetramer. (**C**) Scatterplot of green versus red trichromatic coordinates (TCCs). For visualization purposes, the red TCC coordinates were nudged for each group by a constant value. Blocking experiments were done with 10 mg ml^−1^ mannan (man) or 2 mM EDTA. nd, not determined.

Last, we wondered whether ligand clustering on *MTBC* mycobacteria might induce local recruitment of DC-SIGN. To test this, we used FRET, which relies on the nonradiative energy transfer between a donor and acceptor fluorophore and presents extreme sensitivity to monitor minute changes in distances between the two molecules when in proximity, <~10 nm (Fig. 6, A and B). We made use of our HEK cell line coexpressing enhanced green fluorescent protein (EGFP)– and DsRed-fused DC-SIGN (HEK_EGFP–DC-SIGN/DsRed–DC-SIGN_) and confocal microspectrofluorimetry (*73*), which relies on FRET measurements taken locally at the plasma membrane (~1-μm^2^ zone) of a single cell. Cells were scored as FRET^+^ according to established criteria (fig. S8) (*73*). Incubation of the HEK_EGFP–DC-SIGN/DsRed–DC-SIGN_ cells with *M. bovis* BCG rendered the cells FRET positive in an MOI-dependent fashion, with 54% of the cells scoring as FRET^+^ at MOI 20 (Fig. 6C). This effect was blocked by the addition of mannan or EDTA. In sharp contrast, only 4% of HEK_EGFP–DC-SIGN/DsRed–DC-SIGN_ cells exposed to *M. smegmatis* at MOI 20 showed a FRET shift. These results lead us to conclude that *M. bovis* BCG adhesion to HEK_DC-SIGN_ cells induces the local recruitment of mDC-SIGN, most likely via ligand clustering on the bacterial cell surface, therefore explaining selective and efficient attachment of *MTBC* species to DC-SIGN.

## DISCUSSION

**Fig. 7.**
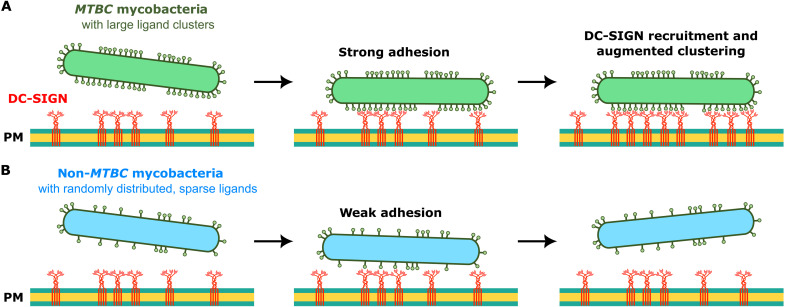
Molecular basis for the selective binding of pathogenic *MTBC* mycobacteria by DC-SIGN. (**A**) Glycoconjugate ligands in high-density nanoscale clusters on *MTBC* mycobacteria surfaces bind DC-SIGN clusters on host immune cells, stimulating further recruitment and clustering of the receptor. (**B**) On the other hand, non-*MTBC* mycobacteria expose DC-SIGN ligands randomly and less densely precluding efficient binding to DC-SIGN on host immune cells. While the enhanced cluster-induced adhesion of *MTBC* mycobacteria will help them to resist mechanical stress (e.g., from hydrostatic pressures), we expect that the weaker nonclustered adhesion of non-*MTBC* mycobacteria will lead to their rapid detachment. PM, plasma membrane.

Hijacking DC-SIGN expressed at the surface of antigen-presenting DCs represents an escape mechanism for several important pathogens (*74*). Therefore, a thorough understanding of the molecular bases of efficient ligand binding leading to bacterial recognition is critical. Here, we show that, beyond ligand specificity, selective and efficient adhesion of pathogenic mycobacteria to host cell membrane DC-SIGN relies on the nanoscale clustering of glycoconjugate ligands on the bacterial cell surface and on adhesion-induced recruitment of the receptor (summarized in Fig. 7).

Binding strength and kinetics of single DC-SIGN–ligand complexes is similar for both pathogenic *M. bovis* BCG (an *MTBC* species) and nonpathogenic *M. smegmatis* (a non-*MTBC* species). However, the distribution of surface-exposed ligands strongly differs between the two species, with dense ligand nanoclusters being observed only on *M. bovis* BCG (Fig. 7). As *Mtb* has coevolved with its human host to evade the immune system (*75*, *76*), it is tempting to speculate that the organization of DC-SIGN ligands into dense clusters has provided an evolutionary advantage for *MTBC* pathogens.

Until recently, nanoscale heterogeneities on living microbial cells were not accessible to study. However, the emergence of live-cell nanoscopy (*45*) has revolutionized the way microbiologists explore the constituents and machineries of living bacteria to molecular resolution. Whereas super-resolution fluorescence nanoscopy enables to study the dynamics of biomolecules and particles inside cells, AFM is capable of imaging and force probing single-cell surface components. In the past years, there have been exciting discoveries demonstrating that many specific molecules such as receptors or ligands on pathogen surfaces are not distributed homogenously but form nanodomains [reviewed in (*77*, *78*)]. Early single-molecule AFM studies with mycobacteria found that the *M. tuberculosis* heparin-binding hemagglutinin adhesin is segregated within nanodomains on the bacterial cell surface (*79*), while its sulphated proteoglycan ligands were distributed homogenously on pneumocytes (*80*). More recently, nanoclustering of various staphylococcal adhesins has been reported, suggesting that they use this phenomenon to favor enhanced, multivalent interactions with host extracellular matrix proteins such as fibrinogen (*81*–*84*). Using hydrophobic tips, we found that a smooth variant of the non-*MTBC* pathogen *Mycobacterium abscessus* exhibits hydrophilic and hydrophobic nanodomains associated with glycopeptidolids (*46*, *85*). Surface compartmentalization of rough lipopolysaccharide classes has also been reported for *Brucella abortus* (*86*). Fungal pathogens also form cell surface nanodomains, as demonstrated for the cell-wall adhesion protein Als5 from *Candida albicans* (*87*). Pulling on single adhesins with AFM tips functionalized with specific antibodies induced the formation of Als5 domains of 100 to 500 nm, resulting from force-induced conformational changes in the protein, and the domains were shown to propagate over the entire cell surface.

An important unsolved question is how clusters are formed on bacterial surfaces. Nano-/micro-domains within bacterial membranes is an emerging, fast-moving field, and their origins, compositions, and functional roles in bacterial physiology and pathology are being unraveled (*88*–*90*). Yet, in many species including mycobacteria, more external envelope layers mask the inner and outer membranes, and, to our knowledge, practically nothing is known about nanodomains within these outermost surface layers where interactions with the host most likely occur. Unraveling how these domains are formed, and how they change over time and in response to chemical or mechanical stresses, constitutes an exciting untapped field of research.

Another key finding is that the clustering of DC-SIGN on the host cell membrane also contributes to efficient and selective binding of *M. bovis* BCG and that adhesion of the latter stimulates local recruitment of the receptor (Fig. 7). This recruitment may involve passive diffusion of DC-SIGN where receptor molecules binding a bacterium are retained, leading to an increase in their local density. However, a mechanism involving microtubules for the rapid, directed transport of DC-SIGN clusters was recently reported and was proposed to bring bound pathogens on the periphery or projections of DCs toward the perinuclear region for internalization (*91*). Future work may explore the mechanism underlying adhesion-dependent recruitment of DC-SIGN.

We speculate that clustering of pathogen ligands and of DC-SIGN host receptors might be a general mechanism for activating pathogen recognition and internalization by antigen-presenting DCs. Recruitment at the adhesion site was reported for zymosan particles derived from the fungal pathogen-surrogate, *Saccharomyces cerevisiae* (*92*), although it remains to be investigated whether binding of fungal pathogens, such as *C. albicans*, to DC-SIGN (*93*) involves ligand clustering or DC-SIGN recruitment. A detailed understanding of this phenomenon could open new avenues in therapeutics, e.g., immunomodulatory or anti-adhesive antimicrobial strategies.

Collectively, our work sheds light on the importance and complexity of surface distribution of both ligands and DC-SIGN in binding of pathogens by this receptor through high-avidity interactions. Recent progress in the development of DC-SIGN antagonists includes multivalent glycomimetic modulators showing great promise (*40*, *94*–*101*).

## MATERIALS AND METHODS

### Bacterial strains and growth conditions

*M. bovis* BCG (Pasteur strain, for microplate and flow cytometry binding experiments; GL2 strain, for AFM and SMFS experiments), *M. smegmatis* mc^2^ 155, *M. chelonae* A6, *M. kansasii* [clinical isolate; (*21*)], and *M. avium* [clinical isolate; (*21*)] were cultured as surface pellicles at the appropriate temperature in Middlebrook 7H9 medium (Sigma-Aldrich) supplemented with 0.1% (w/v) glycerol, 0.1% (w/v) d-glucose, sodium chloride (0.425 g liter^−1^), catalase (2 mg liter^−1^), and bovine serum albumin fraction IV (BSA; 2.5 g liter^−1^). Mature surface pellicles were washed in 7H9 medium (not supplemented with BSA or catalase) and gently dispersed using 3-mm-diameter glass beads yielding suspensions containing single bacterial cells (as observed by microscopy and AFM). Freshly preparedsingle-bacterium suspensions werestained (or not) with the green fluorescent dye fluorescein isothiocyanate (FITC) following a described (*102*) protocol with the exception that buffer solutions contained no detergent. These suspensions were either used immediately for binding experiments or snap frozen in liquid nitrogen and stored at −80°C until their use. For SMFS experiments, an aliquot was thawed, appropriately diluted in nonsupplemented 7H9 medium and seeded in a hydrophobic nontreated polystyrene culture dish (35 mm). After 30-min incubation at 37°C, nonadherent bacteria were washed away, and fresh medium was added before incubation at 37°C for an additional 30 min (this ensured strong immobilization of the bacterial cells). The bacteria were then washed with phosphate-buffered saline (PBS) and the petri dish filled with PBS supplemented with 0.1% BSA, 1 mM CaCl_2_, and 1 mM MgSO_4_. For SCFS experiments, an aliquot was thawed, diluted appropriately in PBS, and seeded on a treated (hydrophilic) polystyrene dish. Bacteria were allowed to settle down and weakly adhere to the surface before they were caught with a hydrophobic beaded AFM cantilever (see SCFS section below).

### Production of recombinant C-type lectins

The coding sequence of the extracellular portion of the DC-SIGN protein (amino acids 64 to 404, including both the helical neck domain and the C-terminal C-type lectin domain) was cloned into the vector pET19b (Novagen). This plasmid was then transformed into *E. coli* BL21 (DE3)plysS, which served as expression strain (*40*). Recombinant protein expression was induced in exponential cultures (optical density at 600 nm = ~0.6) with 1 mM isopropyl-β-D-thiogalactopyranoside (Fluka). After 4 hours of induction at 37°C, the bacteria were collected by centrifugation and the pellets were frozen. They were then thawed, resuspended in lysis buffer [100 mM NaCl, 50 mM tris-HCl, and 0.1% (v/v) Triton X-100 (pH 7.8)], and probe sonicated in a bath of melting ice (three cycles of 30 s). The protein, which was expressed insolubly in inclusion bodies, was collected by centrifugation (45,000*g*, 30 min, 4°C), and the pellet washed twice with washing buffer [1 M NaCl, 25 mM tris, and 1 M urea (pH 7.8)], with intermittent sonication steps. After the last wash, the pellet was resuspended in solubilization buffer [10 mM tris (pH 7.8), 100 mM NaH_2_PO_4_, 6 M guanidine, and 0.01% (v/v) β-mercaptoethanol], and the solution was sonicated (two cycles of 30 s) and ultracentrifuged (55,000*g*, 30 min, 4°C). The recovered supernatant was mixed with nickel–nitrilotriacetic acid (Ni-NTA) agarose with stirring overnight at 4°C before loading the suspension onto a column. After a wash step [with 50 ml of 30 mM tris-HCl buffer containing 1 M NaCl, 1 mM CaCl_2_, 6 M urea, and 15 mM imidazole (pH 7.8)], the protein was renatured on the Ni-NTA agarose column by successive passages with 30 ml of 30 mM tris-HCl buffer (pH 7.8) containing 1 M NaCl and decreasing concentrations of urea (from 5 to 1 M) at a flow rate of 15 ml hour^−1^. The refolded, pure protein was eluted with 30 mM tris-HCl buffer (pH 7.8) containing 1 M NaCl, 1 mM CaCl_2_, and 1 M imidazole, and 10 to 15 1-ml fractions were recovered. The most concentrated fractions were pooled and dialyzed twice overnight at 4°C against 30 mM tris buffer (pH 7.8) containing 1 M NaCl and 1 mM CaCl_2_. The purity and correct tetrameric quaternary state of the produced protein were verified by denaturing and nondenaturing polyacrylamide gel electrophoresis (fig. S2, A and B).

Recombinant C-type lectins used in SMFS experiments (DC-SIGN, Dectin-2, and Mincle) consisted of their C-terminal extracellular domains (amino acids 59 to 404 for DC-SIGN, amino acids 42 to 209 for Dectin-2, and amino acids 41 to 219 for Mincle) fused at their N termini to the C terminus of the human IgG1-Fc1 fragment. All these Fc-lectin fusions were produced in Chinese hamster ovary cells and obtained in purified form (in PBS) from Invivogen (available on request). The predicted maximum extended length of the polyethylene glycol (PEG)–linked IgG-Fc–tagged sDC-SIGN is ~50 nm [~12-nm linker, ~6-nm IgG-Fc (*103*); ~32-nm sDC-SIGN (*104*)], while for both sMincle and sDectin-2, it is ~20 nm [with soluble extracellular domains of ~4 nm; (*105*, *106*)]. However, the linker is expected to attach to lysine residues randomly. In addition, molecules may be attached off-center at the tip with an apex diameter of ~20 nm that may give rise to rupture lengths that underestimate the maximum molecular complex extended length.

### Microplate binding assays using wells coated with *M. bovis* BCG ManLAM

Functionality of purified sDC-SIGN (*40*) was confirmed by an adhesion test between the protein and different mycobacterial ligands using an enzyme-linked immunosorbent assay test (fig. S2, C and D). For this, 100 ng of *M. bovis* BCG ManLAM in an ethanol/water mixture was adsorbed in the wells of a 96-well plate and the ethanol/water was evaporated under a hood. After two washes with tris-buffered saline (TBS)–1 mM CaCl_2_–0.01% Tween 20–1% BSA [tris-buffered saline, 50 mM tris-HCl (pH 7.5), 150 mM NaCl supplemented with 1 mM CaCl_2_, 0.01% (v/v) Tween 20, and 1% (m/v) BSA], the wells were blocked with TBS–1 mM CaCl_2_–5% BSA for 2 hours. After three washes, different quantities of the sDC-SIGN protein were diluted in TBS–1 mM CaCl_2_ or TBS–5 mM EDTA (TBS supplemented with 5 mM EDTA) and added to the wells containing ManLAM. For blocking experiments (fig. S2D), the sDC-SIGN solution was preincubated with various mycobacterial ligands at various concentrations in TBS–1 mM CaCl_2_ for 2 hours at room temperature (RT) before adding it to the wells containing ManLAM. After three washes in TBS–1 mM CaCl_2_–0.01% Tween 20–1% BSA, 100 μl of anti–His-Tag primary antibody (Sigma-Aldrich) diluted 1:3000 in TBS–1 mM CaCl_2_–1% BSA was added to each well for 2 hours. After three washes with TBS–CaCl_2_–1% BSA, 100 μl of mouse anti-Ig secondary antibody coupled to horseradish peroxidase (HRP) (diluted 1:3000 in TBS–CaCl_2_–1% BSA; Sigma-Aldrich) was added to each well for 1 hour. After a final three washes with TBS–1 mM CaCl_2_–1% BSA, HRP was detected using 3,3′,5,5′-tetramethylbenzidine (SureBlue, Eurobio) and 1 M phosphoric acid. The resulting precipitate was read with a spectrophotometer at 450 nm.

### Microplate binding assays using wells coated with mycobacteria

In binding assays using coated mycobacteria, dissociated bacterial preparations (0.5 mg, wet weight) were adsorbed on 96-well microplates (Nunc) in carbonate buffer [15 mM Na_2_CO_3_, 35 mM NaHCO_3_, and 1% (w/v) sodium deoxycholate] overnight at 4°C. Wells were then blocked for 2 hours at 37°C with TBS–2 mM CaCl_2_–10% BSA and extensively rinsed with TBS–2 mM CaCl_2_–1% BSA–1% Tween 20. sDC-SIGN (2 μg ml^−1^ in TBS–2 mM CaCl_2_–1% BSA) preincubated or not with 100 mM mannose and allowed to react with mycobacteria for 2 hour at RT. Wells were washed, and bound sDC-SIGN was detected using mouse monoclonal anti–His-Tag antibody (Sigma-Aldrich) and HRP-conjugated anti-mouse IgG antibody (Sigma-Aldrich). HRP was detected as above.

### Microplate binding assays using wells coated with sDC-SIGN

In binding assays using plate-bound sDC-SIGN, the protein (2 μg ml^−1^ in TBS–2 mM CaCl_2_–His-1% BSA) was allowed to react for 2 hours at RT with Ni-chelate microplates (Nunc). Wells were then blocked and rinsed as indicated above. Dissociated bacterial preparations (0.5 mg, wet weight) or *M. bovis* BCG ManLAM (*107*) (at the indicated amount) in TBS–2 mM CaCl_2_–1% BSA were preincubated or not with 50 or 100 mM mannose or galactose or 1 mM EDTA and allowed the reaction with plate-bound sDC-SIGN for 2 hours at RT. After washing, bound mycobacteria or ManLAM was labeled with HRP-conjugated concanavalin A (Sigma-Aldrich). HRP was detected as above.

### Construction of HEK–DC-SIGN cell lines

Briefly, a cDNA (gift from O. Neyrolles, IPBS, Toulouse) encoding the gene of DC-SIGN/CD209 was amplified by the polymerase chain reaction (PCR) using primers 5′-GATATCTACGTAAGTGACTCCAAGGAACCAAGACTGC-3′ and 5′-GATATCTCTAGACTACGCAGGAGGGGGGTTTGGGGTG-3′. PCR product lacking the initiation codon was then double-digested with Sna BI and Xba I (underlined sequences in primers). The generated segment was inserted into a modified SK+ bluescript plasmid, containing Sna BI–Xba I restriction sites, downstream of an epitope tag from the bacteriophage T7 fused with either EGFP cDNA or DsRed monomer cDNA (Clontech). Fragments named T7-EGFP–DC-SIGN and T7-DsRedmono–DC-SIGN were then cloned into Ecor V–Xba I-digested PcDNA3.1/Hygro or PcDNA3.1/neo vectors (Invitrogen), respectively. The constructs were verified by restriction enzyme analysis and Sanger sequencing.

HEK 293 cells were seeded in Dulbecco’s modified Eagle’s medium (DMEM) plus 10% fetal calf serum in six-well culture plates at a density of 5 × 10^5^ cells per well and were cultured overnight. Cells were approximately 80% confluent at time of transfection. Ten micrograms of PcDNA3.1/EGFP–DC-SIGN, PcDNA3.1/DsRedmono–DC-SIGN, or PcDNA3.1/mock vector DNA was diluted in 400 μl of a solution of 150 mM NaCl. Twenty-five microliters of jetPEI (Polyplus) transfection reagent was diluted in 400 μl of the same solution and added to the plasmid mixture. After a gentle mixing, the DNA:lipid mixture was incubated at RT for 30 min. Two hundred microliters of the mixture was added per well (to a final culture volume of 3 ml per well). Cells were maintained at 37°C and 5% CO_2_ for 24 hours. Double-transfected cells (HEK_EGFP–DC-SIGN/DsRed–DC-SIGN_) were obtained by repeating the same transfection procedure using the EGFP–DC-SIGN–transfected cells and the PcDNA3.1/DsRedmono-DC-SIGN vector DNA.

HEK cells transfected with PcDNA3.1/EGFP–DC-SIGN (HEK_EGFP–DC-SIGN_) and with PcDNA3.1/DsRedmono–DC-SIGN (HEK_DsRed–DC-SIGN_) were respectively selected with hygromycin B (200 μg ml^−1^) and G418 (400 μg ml^−1^). After the cells’ expansion over 4 weeks, transfected cells were sorted by flow cytometry (Beckman Coulter Epics Altra). Adherent cells were recovered by flushing and then incubated with anti–DC-SIGN antibody (0.5 μg ml^−1^; R&D Systems) in PBS containing 0.5% BSA (PBS-BSA) for 30 min at 4°C. The cells were washed twice with cold PBS-BSA and incubated on ice with Alexa Fluor 647–labeled goat anti-mouse Ig. After washing, the cells were sorted for both fluorescent protein and DC-SIGN double-positive signal. Controls, including (i) untransfected cells, (ii) transfected but unstained cells, and (iii) transfected cells stained with isotype control, were used to define the gating strategy for cell sorting. Transfected cells were limiting diluted to the concentration of 5 cells ml^−1^ and plated in 200-μl of medium in 96-well dishes. Cell expansion was done in 24- and 6-well dishes when 80% of confluence was reached in the previous wells. The stable expression of DC-SIGN was confirmed by flow cytometry, as described for cell sorting, on a FACSCalibur flow cytometer (Becton Dickinson, San Jose, CA).

### Binding assay using HEK-DC-SIGN cell lines

Bacteria were first labeled with biotin hydrazide after periodate oxidation (*21*). Around 1 g (wet weight) of bacteria was washed twice with PBS and resuspended in 200 μl of 0.1 M ammonium acetate buffer (pH 5.5) containing 15 mM sodium metaperiodate (Merck). After a 20-min incubation at 4°C in the dark with gentle rotation, the oxidation reaction was quenched by adding 200 μl of 0.1 M ammonium acetate buffer (pH 5.5) containing 30 mM sodium bisulphite (Sigma-Aldrich). After centrifugation, bacteria were resuspended in 400 μl of PBS containing 5 mM biotin hydrazide (Sigma-Aldrich). After a 2-hour incubation at RT with gentle rotation, cells were washed three times with PBS. Biotin-labeled bacteria were resuspended in PBS and added to 2 × 10^5^ HEK cells at the indicated MOI in a total volume of 1 ml of cold DMEM medium. After a 4-hour incubation at 4°C under gentle rotation, the cell suspension was centrifuged at 300*g* for 10 min. The pellet was suspended in 50 μl of allophycocyanin-conjugated streptavidin (BD Pharmingen). After 20 min at 4°C in the dark, cells were washed twice with PBS, resuspended in 400 μl of PBS, and analyzed by confocal microscopy (fig. S9) and flow cytometry using a FACSCalibur CellQuest Pro (BD Biosciences) software.

### AFM tip functionalization for SMFS

For SMFS experiments, we functionalized AFM tips with flexible PEG linkers terminated with a reactive aldehyde (Ald) function allowing the covalent coupling of an IgG-Fc–fused lectin (*56*, *57*). The net negatively charged extracellular domains of the lectins (sDC-SIGN: isoelectric point (pI) 5.2, sDectin-2: pI 5.7, and sMincle: pI 5.1) in PBS (pH 7.4) are repelled by the net negatively charged Ald-PEG–coated tip surface, while IgG1-Fc1 (pI 7.6) is not. Therefore, the IgG1-Fc1 fusion configuration favored the covalent coupling of a primary amine group within N-terminal IgG1-Fc1 and, hence, optimal geometry of the lectin extracellular domain for interactions with probed ligands. D-cantilevers (nominal *k* = 10 pN nm^−1^) of Bruker MSCT silicon nitride AFM probes were used. The AFM probes were functionalized at RT with Fc-lectins using Ald-Ph-PEG_24_-NHS ester (BroadPharm) bifunctional linkers. Briefly, the bare silicon nitride AFM probes were washed with chloroform, dried under nitrogen flow, ultraviolet (UV) ozone–treated, amino-functionalized using the 3-aminopropylyriethoxysilane and triethylamine in the gas-phase method (*57*), and lastly placed in a chloroformic solution of Ald-Ph-PEG_24_-NHS ester (6.6 mg ml^−1^) and triethylamine [6% (v/v)]. After 2 hours, they were washed thoroughly with chloroform, dried under nitrogen flow, and immersed in 50 μl of an Fc-lectin solution [50 μg ml^−1^ in PBS (pH 7.4)] to which 1 μl of sodium cyanoborohydride (1 M stock solution) was added immediately. After 1 hour of incubation, unreacted free Ald groups were quenched through the addition of 2.5 μl of ethanolamine hydrochloride (1 M stock solution, pH 8.0), and the fully functionalized AFM probes were thoroughly washed with PBS buffer. Fc-lectin AFM probes were stored at 4°C and used within 48 hours.

### AFM imaging and single-molecule force spectroscopy

AFM imaging was performed with bare MSCT-D cantilevers in PBS buffer using the Quantitative Imaging mode (approach and retract velocity of 40 μm s^−1^, *z* length of 600 nm for whole bacteria, and 150 nm for high-resolution images). Average roughness values were obtained for second-order polynomial line-leveled, high-resolution images (256 pixels by 256 pixels, 300 nm by 300 nm) recorded on top of bacteria.

All SMFS experiments were carried out in PBS supplemented with 0.1% BSA, 1 mM CaCl_2_, and 1 mM MgSO_4_ using a JPK NanoWizard 4 NanoScience AFM. Force spectroscopy data were collected in force mapping (force-volume) mode using a constant approach and retraction velocity of 1 μm s^−1^, a ramp length of 250 nm, a contact force set point and pause of 250 pN and 250 ms, respectively, a closed z-loop, and fast and slow scan axes of 250 nm (16 pixels) and 1 μm (64 pixels), respectively. For dynamic force spectroscopy (dfs) and contact-time versus binding frequency experiments, respectively, retraction velocity and contact pause were varied as indicated in the relevant figures.

### Single-cell force spectroscopy

Bacterial probes were prepared as previously described with modifications (*108*). Using the AFM, the first ~5 μm of a triangular tipless cantilever (NP-O10, Bruker) was brought into contact with a thin layer of UV light-curable glue (NOA 63, Norland Edmund Optics). The glue-covered part of the cantilever was then brought into contact for 3 min with a silica bead of 6.1 μm diameter (Bangs Laboratories). Afterward, the colloidal probe was taken out of the AFM, and the glue was cured under UV light for 30 min. Colloidal probes were rendered hydrophobic by siliconization. They were placed in a glass dish inside a vacuum chamber along with a separate glass dish containing 100 μl of Sigmacote (Sigma-Aldrich) siliconization reagent, and a vacuum (200 mbar) was applied for 1 hour. Then, they were placed in a 45°C oven for 30 min before being stored in ultrapure water until use. Bacterial probes were made by bringing a hydrophobic colloidal probe in contact (applying 5 nN for 60 s) with a single FITC-stained bacterium (observed through a 40× objective of an inverted epifluorescence microscope) on a tissue culture–treated polymer coverslip bottom dish (iBidi) in PBS. The dish was then exchanged with a similar dish containing HEK cells and gently fixed as follows: Cocultured HEK_DC-SIGN_ and HEK_WT_ cells were first washed three times with PBS supplemented with 1 mM CaCl_2_ and 1 mM MgSO_4_ (PBS^Ca/Mg^) to remove BSA. They were immediately fixed with 4% formaldehyde solution in PBS (Invitrogen) for 15 min at RT, washed (4×, 5 min) with PBS^Ca/Mg^, and stored at 4° to 8°C until use on the same day. SCFS was carried out in PBS^Ca/Mg^ using a constant approach and retraction velocity of 20 μm s^−1^, a ramp length of 1 μm, a contact force set point of 500 pN, no additional pause at contact, a closed z-loop, and a scan area of 3.2 μm by 3.2 μm (32 × 32 pixels) located on the apical portion of a cell. Because their soft membranes and dynamic movements complicated AFM SCFS measurements and because cross-linked sDC-SIGN retains its carbohydrate-binding activity (*24*, *104*), we gently formaldehyde-fixed the HEK cells (*109*).

### Force spectroscopy data analysis

In *FD* curves, the last rupture peak that could be fitted with the worm-like chain model (*110*) of polymer extension was considered as representing the extension and rupture of a single lectin-ligand complex and was used to obtain rupture forces and lengths (see Fig. 2). FD curve analyses were performed using JPK data processing software. DC-SIGN interaction with ligands was approximated with pseudo–first-order kinetics (*67*), allowing the estimation of *k*_on_ according to the formula

$$k_{\mathrm{on}}={\left(\tau c_{\mathrm{eff}}\right)}^{-1}$$

where τ is the interaction time and *c*_eff_ is the effective concentration. τ was determined from a fit of binding frequency (*B*_F_) versus contact time (*T*_c_) data with the following function

$$B_{F}(t)=A\left(1-e^{\frac{T_{c}-T_{0}}{\tau }}\right)$$

*c*_eff_ was calculated using the following formula

$$c_{\mathrm{eff}}=\frac{3n_{b}}{2N_{A}\pi r_{\mathrm{eff}}^{3}}$$

where *n*_b_ is the number of binding pairs (≈1), *N*_A_ is the Avogadro constant, and *r*_eff_ is equal to the radius (in decimeters) of a half sphere whose diameter is equal to the combined approximate equilibrium lengths of the PEG_24_ linker (6 × 10^−8^ dm), the N-ter IgG1-Fc1 fusion (~6.5 × 10^−8^ dm), and the soluble extracellular domain of DC-SIGN (~32 × 10^−8^ dm). For dfs data, the most probable rupture forces (*F*) representing single molecular bonds for five log-equispaced *LR* bins were fit with the Bell-Evans model (*70*)

$$F(LR)=\frac{k_{B}T}{x_{\beta }}\cdot ln\left(\frac{LRx_{\beta }}{k_{B}Tk_{\mathrm{off}}}\right)$$

where *k*_B_ is the Boltzmann constant, *T* is the temperature (≈293 K), *x*_β_ is the distance along the reaction coordinate to the transition between bound and unbound states, and *k*_off_ is the off-rate constant.

### FRET experiments

FRET measurements on single cells were performed using a spectrofluorimeter and analysis procedure described elsewhere (*73*). Briefly, the measuring system consisted of a microscope (Zeiss Axioplan) equipped with a 40× oil immersion objective (numerical aperture of 1.3) and pinholes to improve spatial resolution, an excitation laser line (Coherent Inova 90C), and a spectrograph for fluorescence recording (Horiba Jobin-Yvon Symphony). Fluorescence spectra for GFP/DsRed FRET experiments were recorded from 495 to 700 nm with 0.59-nm spectral resolution upon donor excitation at 476 nm. Recorded fluorescence spectra being different from one cell to the other, depending on levels of dye expression and the autofluorescence contribution, were converted into trichromatic coordinates according to the CIE 1931 international standard (*111*). These coordinates from recorded spectra allowed their classification for those cells that express only the donor or the acceptor and for those that express variable amounts of FRET.

### Statistical analyses

All statistical analyses were conducted using the R programming language, and graphs were drawn in RStudio. Sample sizes and replicates are reported in figure legends. Experiments were repeated a minimum of two times.

## Notes

View/request a protocol for this paper from *Bio-protocol*.
