# Supplementary material for: Nanoscale clustering of mycobacterial ligands and DC-SIGN host receptors are key determinants for pathogen recognition
Source: Sci Adv. 2023 May 19;9(20):eadf9498. doi: 10.1126/sciadv.adf9498 (PMC10198640; doi:10.1126/sciadv.adf9498)
Supplement: Supplementary file 1 — Figs. S1 to S9 Legend for data S1 [file sciadv.adf9498_sm.pdf]

Science Advances
AAAS

Supplementary Materials for

Nanoscale clustering of mycobacterial ligands and DC-SIGN host receptors are key determinants for pathogen recognition

Albertus Viljoen *et al.*

Corresponding author: Yves F. Dufrêne, yves.dufrene@uclouvain.be; Jérôme Nigou, jerome.nigou@ipbs.fr

Sci. Adv. **9**, eadf9498 (2023)
DOI: 10.1126/sciadv.adf9498

The PDF file includes:

Figs. S1 to S9
Legend for data S1

Other Supplementary Material for this manuscript includes the following:

Data S1

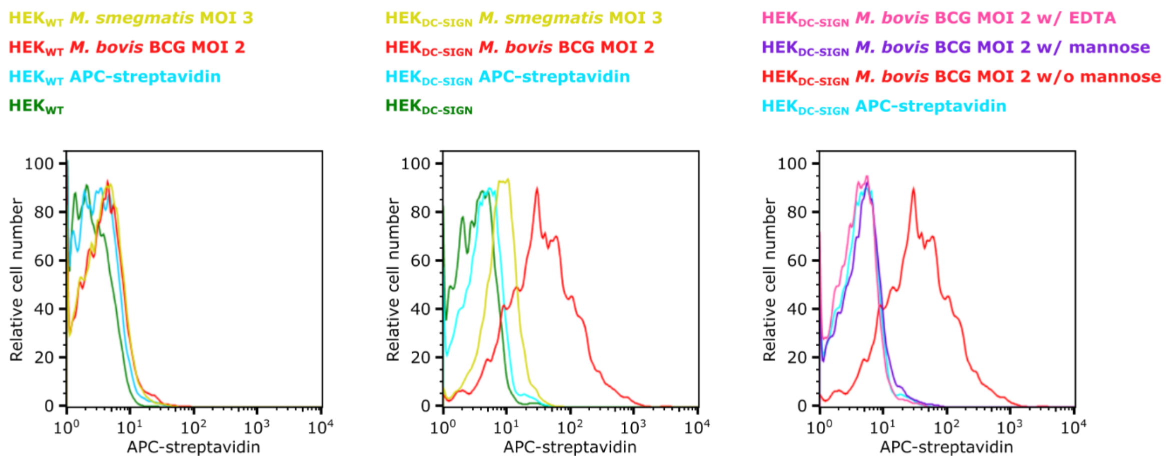

Fig. S1. Raw flow cytometry data of allophycocyanin (APC)-conjugated streptavidin-labelled mycobacteria binding HEK cells.

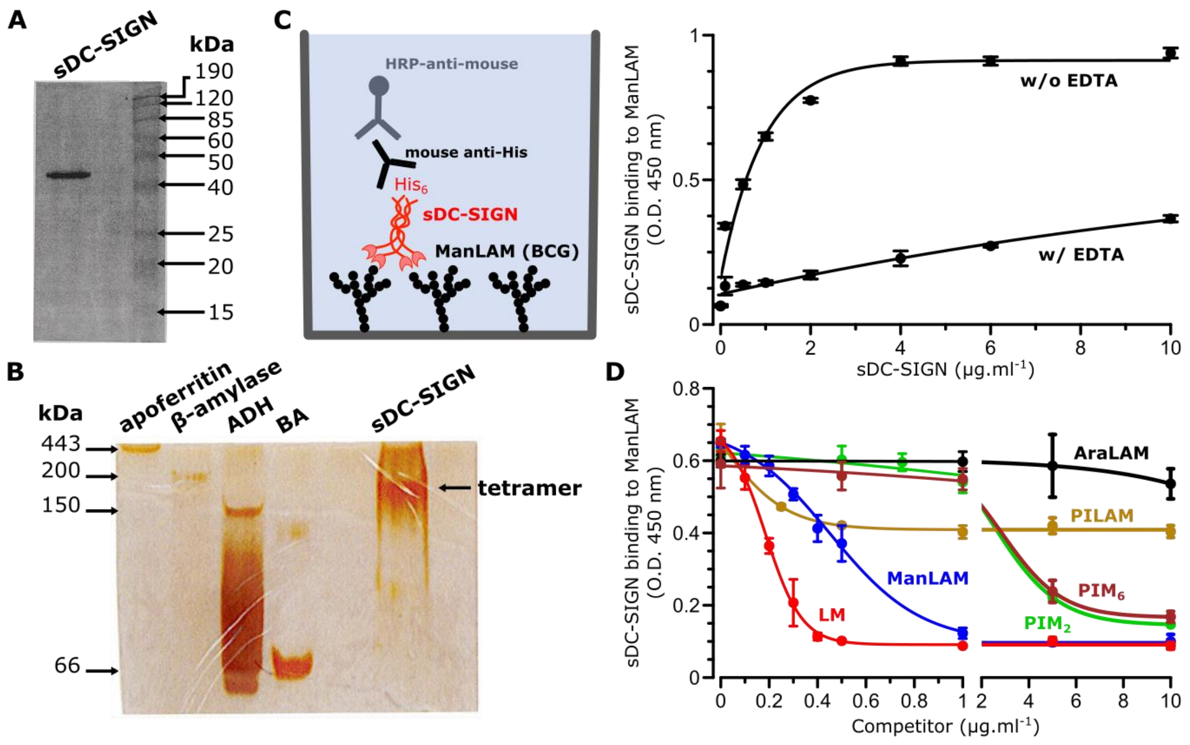

Fig. S2. Purity, oligomeric state and functionality assessment of purified sDC-SIGN. (A, B) SDS-PAGE (A) and native PAGE (B) gels showing high purity and correct tetrameric state of sDC-SIGN, respectively. ADH, alcohol dehydrogenase; BA, bovine albumin. (C) ELISA assay with *M. bovis* BCG ManLAM-coated (100 ng per well) microwell plates showing that purified tetrameric sDC-SIGN is functional. EDTA 5 mM blocks binding. (D) Inhibition of sDC-SIGN (1 µg.ml^{-1}) binding to *M. bovis* BCG ManLAM-coated microwells by ligands present in *M. bovis* BCG and *M. smegmatis*. LM, lipomannan; PIM_2, phosphatidylinositol dimannoside; PIM_6, phosphatidylinositol hexamannoside; PILAM, *M. smegmatis* phosphoinositol-capped LAM, AraLAM, *M. chelonae* uncapped LAM. LM, PIM_2 and PIM_6, are present in both species. ManLAM is present in *M. tuberculosis* complex species like *M. bovis* BCG, while PILAM is present in *M. smegmatis*.

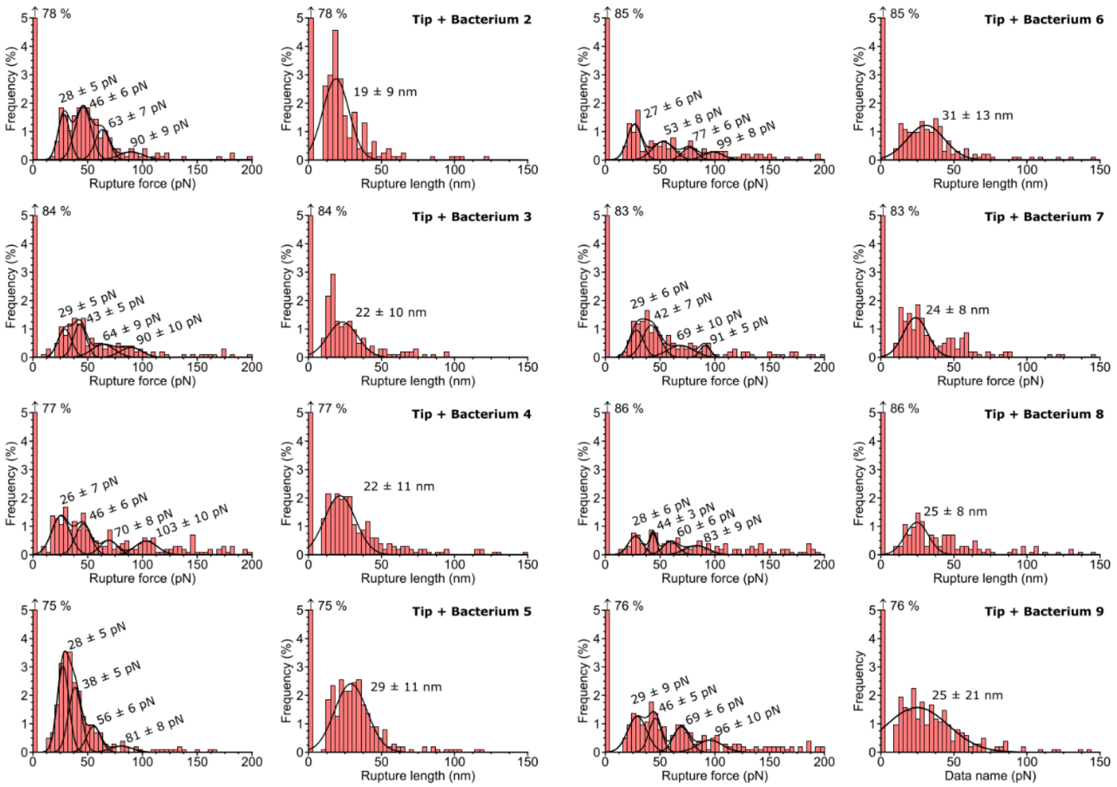

Fig. S3. Rupture force and length histograms obtained with independent sDC-SIGN-modified tips and *M. bovis* BCG bacteria. A total of 1,024 *FD* curves were used to plot each set of histograms. Mean ± standard deviation (s.d.) is indicated above the gaussian fits of the histograms.

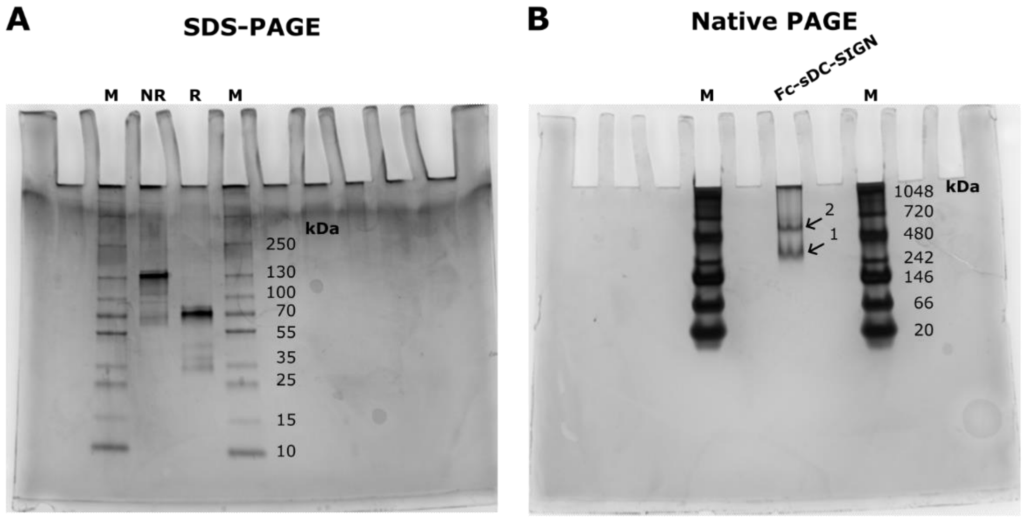

Fig. S4. Oligomeric state of Fc-sDC-SIGN. (A) Silver-stained SDS-PAGE gel showing dimeric (through a disulfide bridge) denatured Fc-sDC-SIGN under non-reductive conditions (NR, 138 kDa) and monomeric Fc-sDC-SIGN under reductive conditions (R, 69 kDa). The molecular weight marker (M) is PageRuler prestained protein ladder from Thermo Scientific (26616). (B) Silver-stained native PAGE gel showing Fc-sDC-SIGN mainly in tetrameric (1, 276 kDa) and di-tetrameric (2, 552 kDa) forms. The latter possibly consists of two tetramers joined through disulfide bridges between Fc chains in the two tetramers. No monomeric, dimeric, or tri-tetrameric forms are visible. The molecular weight marker (M) is NativeMark Protein standard from Invitrogen (LC0725).

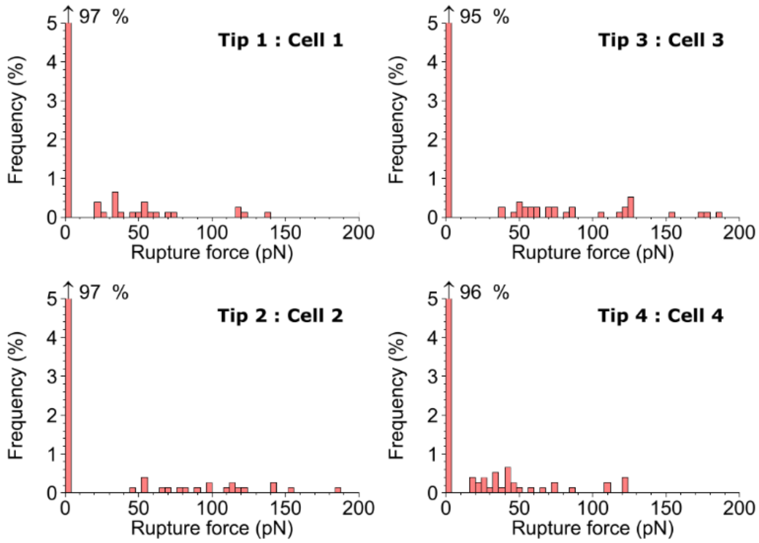

Fig. S5. Rupture force histograms showing that non-functionalized bare tips do not bind *M. bovis* BCG. A total of 1,024 FD curves were used to plot each histogram.

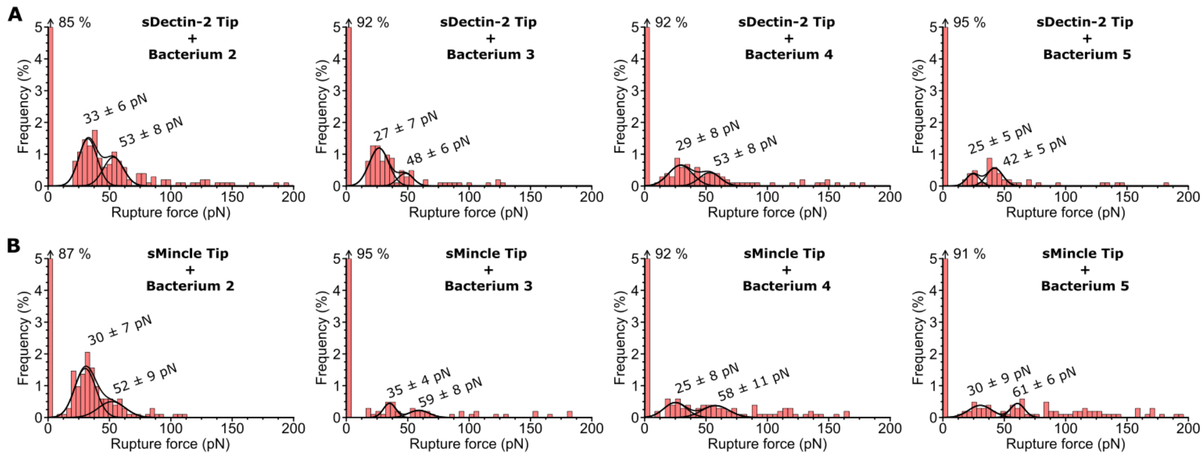

Fig. S6. Multiple force populations observed for C-type lectin-modified tips used to probe *M. bovis* BCG bacteria. Rupture force histograms obtained with independent (A) sDectin-2-modified or (B) sMincle-modified AFM tips and *M. bovis* BCG bacteria. A total of 1,024 *FD* curves were used for each histogram plot. Mean ± standard deviation (s.d.) is indicated above the gaussian fits of the histograms.

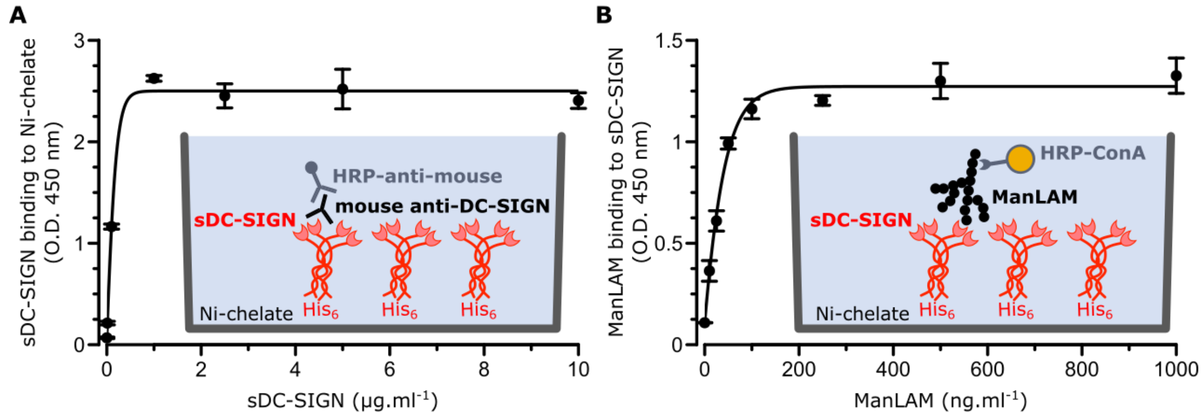

Fig. S7. Controls demonstrating high-density coating (A) and functionality (B) of sDC-SIGN on microtiter plates. (A) sDC-SIGN bound to Ni-chelate microtiter plates was detected by mouse anti DC-SIGN primary antibodies and anti-mouse IgG-HRP secondary antibodies. (B) Binding of ManLAM to sDC-SIGN (2 μg.ml^{-1}) immobilized on the Ni-chelate plates. ManLAM was detected by ConA-HRP.

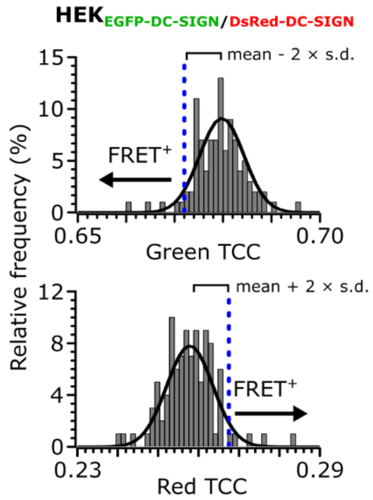

Fig. S8. Evaluating FRET positivity. Cells were scored as FRET$^+$ when green TCC values were less than the mean minus 2 times the standard deviation (s.d.) of values measured for untreated HEK$_{\text{EGFP-DC-SIGN/DsRed-DC-SIGN}}$ and red TCC values were greater than the mean plus 2 times the s.d. of values measured for these cells.

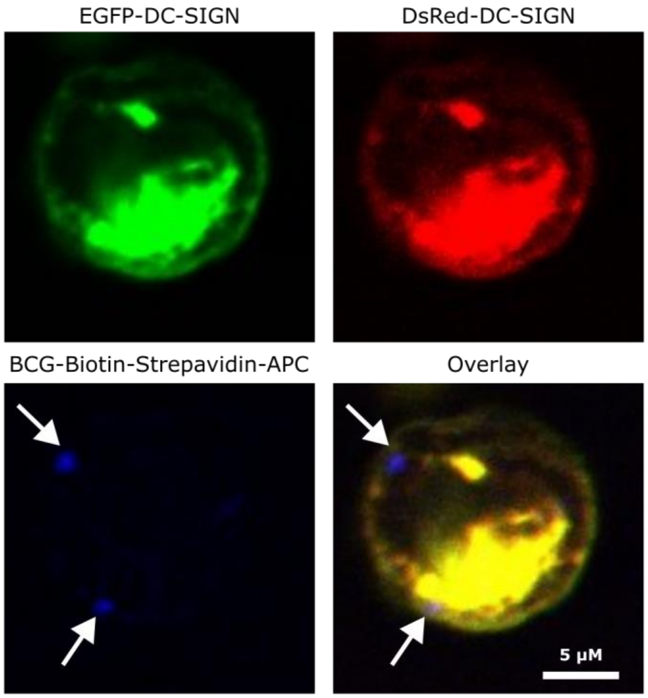

Fig S9. Confocal images of streptavidin-APC-stained biotinylated *M. bovis* BCG bacteria bound to HEK$_{\text{EGFP-DC-SIGN/DsRed-DC-SIGN}}$ cells.

Data S1. (separate file)

Zip archive containing raw data files (excel or tab-separated values) used to draw each figure.
